# Preliminary study on the intraoperative application of the “dual-path” strategy for sentinel lymph node tracing in endometrial cancer

**DOI:** 10.1038/s41598-026-48295-8

**Published:** 2026-05-11

**Authors:** Fangyi Huang, Xinpeng Yao, Ruyu Shao, Desheng Yao, Hooman Soleymani Majd, Yousheng Wei

**Affiliations:** 1 Department of Gynecologic Oncology, Guangxi Medical University Cancer Hospital, Guangxi Zhuang Autonomous Region, Nanning, 530021 P.R. China; 2 Department of cancer and Genomic sciences, College of Medicine and Health, University of Birmingham GB, Birmingham, UK; 3 Department of Gynaecology Oncology, Oxford University Hospitals NHS Trust, Oxford, UK

**Keywords:** Lymphatic pathway of infundibulopelvic ligament, Sentinel lymph node, Endometrial cancer, Cancer, Diseases, Medical research, Oncology

## Abstract

At present, in endometrial cancer surgery, sentinel lymph node tracing via cervical tracer injection is mainly used to assess the metastatic status of pelvic lymph nodes, but it is difficult to evaluate the metastatic status of para-aortic lymph nodes. This study aimed to explore the feasibility of tracing pelvic and para-aortic sentinel lymph nodes drained through different pathways by injecting different tracers via the cervix and proper ovarian ligament respectively during endometrial cancer surgery.A total of 32 patients with endometrial cancer who underwent laparoscopic surgery at the Affiliated Cancer Hospital of Guangxi Medical University from January 2024 to January 2026 were enrolled as the research subjects. Methylene blue solution was injected via the cervix to visualize pelvic sentinel lymph nodes, while indocyanine green (ICG) was injected via the proper ovarian ligament to visualize para-aortic sentinel lymph nodes. The visualization status of sentinel lymph nodes was observed.All sentinel lymph nodes (SLNs) visualized by cervical injection of methylene blue were confined to the pelvic cavity: 92% of lymphatic drainage followed the upper paracervical pathway (UPP), with SLNs located in the obturator and external iliac regions; 8% followed the lower paracervical pathway (LPP), with SLNs in the common iliac region; no infundibulopelvic ligament pathway (IPP) was detected.ICG injection via the proper ovarian ligament visualized SLNs draining through the IPP. The time to initial SLN visualization ranged from 10 s to 137 s (median, 45 s; mean, 50.9 s). Left-sided SLNs were situated in the para-aortic region between the inferior mesenteric artery and left renal vein, with a visualization rate of 80.6% and an accuracy of 95.8%. Right-sided SLNs were located on the inferior vena cava surface 2–3 cm above the aortic bifurcation, with a visualization rate of 93.8% and 100% accuracy. There was no overlap between the visualization pathways of methylene blue and ICG. One case of postoperative lymphatic fistula occurred. The “dual-path” strategy—using different tracers injected via the cervix and proper ovarian ligament to visualize pelvic and para-aortic SLNs respectively—is feasible in endometrial cancer surgery, enabling more comprehensive and accurate assessment of lymph node metastatic status.

**Supplementary Information:**

The online version contains supplementary material available at 10.1038/s41598-026-48295-8.

## Introduction

In the lymph node metastasis pattern of endometrial cancer, isolated or skip metastasis of para-aortic lymph nodes (PANs) carries significant clinical implications. Studies have demonstrated a relatively high proportion of isolated para-aortic lymph node metastasis (i.e., metastasis without pelvic lymph node involvement, termed skip metastasis) among patients with para-aortic lymph node involvement. For example, one study reported that 42 cases (21%) out of 200 patients with pathologically staged IIIC early-stage endometrial cancer postoperatively presented with isolated para-aortic lymph node metastasis, indicating that such metastasis is not uncommon.Furthermore, the risk of isolated para-aortic metastasis rises significantly when the tumor is located in the uterine fundus (fundal cavity or myometrium) or anterior wall myometrium^1^. In contrast, concurrent pelvic and para-aortic lymph node metastasis also accounts for a considerable proportion^2^, suggesting that the conventional pelvic drainage-focused lymph node assessment strategy may miss upper-level metastatic lesions. This underscores the relatively high incidence of para-aortic lymph node metastasis in endometrial cancer, particularly the risk of skip metastasis associated with specific tumor locations.

Systematic lymphadenectomy for early-stage high-risk endometrial cancer may cause overtreatment and unnecessary surgical complications^3^; conversely, low-risk or early-stage endometrial cancer still carries a notable risk of lymph node metastasis—for example, the incidence of lymph node metastasis in POLEmut ECs patients can be as high as 14.2%^4^—thus, sentinel lymph node biopsy (SLNB) is considered a more precise approach for all subtypes of early-to-mid-stage endometrial cancer, as cervical tracer injection has now become the mainstream method for SLNs mapping in endometrial cancer, widely adopted in clinical practice and recommended by multiple international guidelines^5^, which improves the detection rate of lymph node metastasis while reducing surgical complications^6^; despite the remarkable advances of this technique, several key challenges remain: cervical tracer injection requires the tracer to travel a long pathway to reach para-aortic lymph nodes, which is not only time-consuming but also liable to result in uncertain visualization due to the complexity and individual variability of lymphatic drainage, and this approach primarily visualizes pelvic SLNs, making it difficult to assess the metastatic status of para-aortic lymph nodes, so resecting only positive pelvic SLNs may lead to missed detection of positive para-aortic lymph nodes, thereby misclassifying patients who should receive more aggressive treatment (e.g., extended-field radiotherapy) into the low-risk group; on the other hand, patients with tumors located in the uterine fundus or cornua face a higher risk of skip metastasis to para-aortic lymph nodes, and the SLNs visualized via cervical injection may not be the true sentinel nodes, therefore, when SLNs are located in upper regions such as the para-aortic area, the traditional cervical tracer injection method may fail to reliably identify the correct SLNs, necessitating further technical optimization or combination with other mapping modalities to enhance its reliability.

**Fig. 1:**
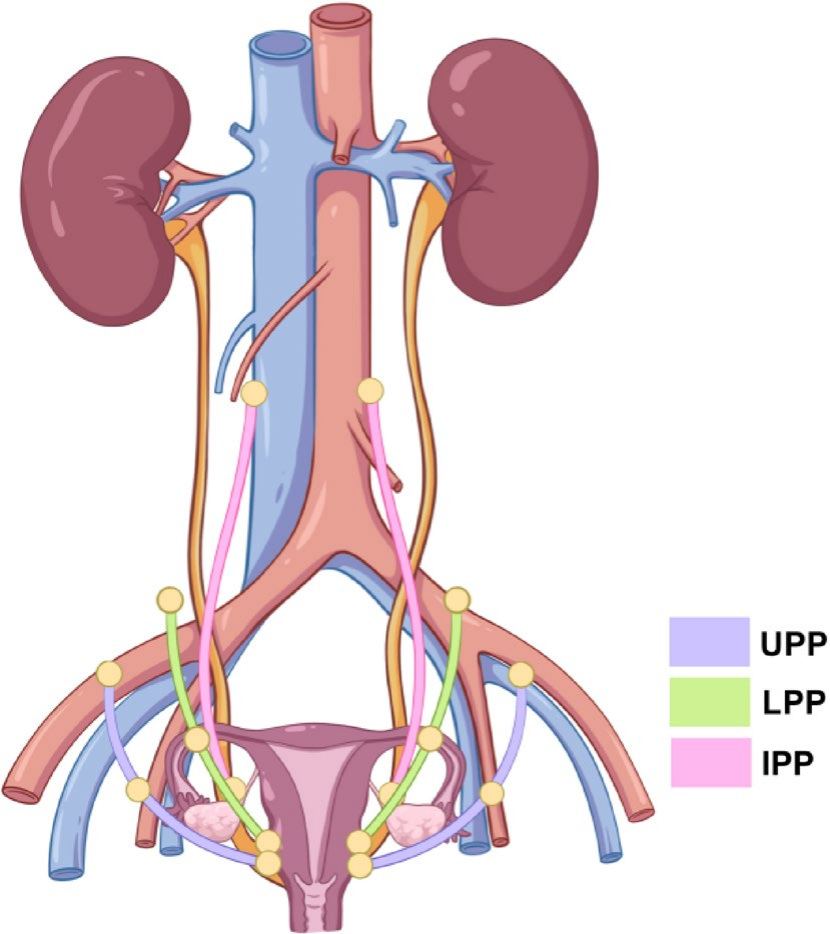
Infundibulo pelvic pathway, IPP^8^.

Studies have shown that there are three lymph node metastasis pathways in endometrial cancer. The most common lymphatic drainage pathway of SLNs from uterine tumors is the upper paracervical pathway (UPP), where the lymphatic trunks of the uterine corpus usually cross the obliterated umbilical artery, and pelvic SLNs are most frequently located medial to the external iliac artery or in the upper part of the obturator fossa region. Another rare pathway is the lower paracervical pathway (LPP), in which lymphatic vessels do not cross the obliterated umbilical artery but run cephalad along the mesentery of the mid-ureter; in this case, SLNs are commonly found in the common iliac and presacral regions^7^. There is also an often-overlooked pathway called the infundibulopelvic pathway (IPP), where lymphatic vessels extend along the infundibulopelvic ligament to the para-aortic lymph node region (Fig. 1)^8^. Therefore, injection of ICG contrast agent into the infundibulopelvic ligament enables tracing of para-aortic sentinel lymph nodes.

Currently, the main methods for tracing the infundibulopelvic ligament pathway involve tracer injection into the uterine fundus or directly into the infundibulopelvic ligament. The former yields an acceptable visualization rate but low accuracy^9^, while the latter is prone to causing bleeding. In this study, during endometrial cancer surgery, we innovatively injected a tracer into the proper ovarian ligament to visualize para-aortic sentinel lymph nodes via the IPP. Meanwhile, a different tracer was injected into the cervix to visualize pelvic sentinel lymph nodes via the UPP or LPP, thereby enabling comprehensive and accurate assessment of sentinel lymph nodes in endometrial cancer. Herein, we report the relevant findings.

## Materials and methods

### General information

A total of 32 patients with endometrial cancer admitted to the Affiliated Cancer Hospital of Guangxi Medical University from January 2024 to January 2026 were enrolled. The patients were aged 25 to 70 years, with a median age of 57 years. According to the FIGO 2023 staging system, their preoperative clinical stages were from stage ⅠA to Ⅱ. All patients were pathologically diagnosed with endometrial cancer prior to surgery and scheduled to undergo extrafascial total hysterectomy or radical hysterectomy combined with pelvic sentinel lymph node exploration and biopsy plus para-aortic sentinel lymph node exploration and biopsy, with or without pelvic lymphadenectomy and para-aortic lymphadenectomy. Histological types included 30 cases of endometrioid adenocarcinoma, 1 case of clear cell carcinoma, and 1 case of carcinosarcoma. Exclusion criteria were as follows: history of cervical treatment, radiological evidence of obvious retroperitoneal lymph node metastasis or distant metastatic lesions, preoperative radiotherapy, severe tubal adhesion, and severe pelvic or abdominal adhesion.

All participants provided written informed consent prior to enrollment. This study was approved by the Institutional Ethics Committee of the Affiliated Cancer Hospital of Guangxi Medical University (Ethics Approval No.: KY20251100). The study was conducted in accordance with the Declaration of Helsinki and Good Clinical Practice guidelines.

### Methods

#### Surgical methods

Using an Optomind 4 K fluorescent laparoscope, methylene blue was injected into the cervix after the trocars were inserted into the abdominal cavity, followed by indocyanine green (ICG) injection into the proper ovarian ligaments. The detailed procedure was as follows: a 1-mL disposable sterile syringe was used to slowly inject 1 mL of methylene blue solution (Jichuan Pharmaceutical, 2 mL:20 mg) at each of the 3- and 9-o’clock positions of the cervix, with both deep (1 cm) and superficial (5 mm) layers, for a total volume of approximately 2 mL. Under laparoscopic visualization, the proper ovarian ligaments were fully exposed. A disposable sterile intravenous infusion set (0.55 × 19 mm) was introduced into the pelvic cavity through a trocar, and approximately 1 mL of diluted ICG solution was slowly injected into the exposed ligament at a depth of 1–2 mm.The ICG agent used was indocyanine green for injection (National Medical Products Agency Approval No. H20055881) manufactured by Dandong Yichuang Pharmaceutical Co., Ltd., with a specification of 25 mg per vial. The dilution procedure was: one vial of ICG (25 mg) was dissolved in 200 mL of sterile water for injection with thorough vortex mixing to prepare a working solution of 0.125 mg/mL. The solution was prepared fresh immediately before use, stored in a light-proof container, and all injections were completed within 2 h to avoid reagent degradation by light that could impair fluorescence. This concentration produced stable and clear fluorescent signals under the Optomind 4 K fluorescent laparoscope used in this study, which optimally met the imaging requirements for intraoperative para-aortic lymph node mapping and ensured the accuracy and reliability of the experimental data.Gentle local compression was applied after injection to prevent drug extravasation, and laparoscopic gauze was placed around the injection site to absorb any leaked ICG and avoid nonspecific staining that might obscure the surgical field. The time from injection initiation was recorded, and the first nodes to demonstrate fluorescence or blue staining were defined as sentinel lymph nodes (SLNs). Under direct laparoscopic visualization, the course of stained lymphatic vessels was tracked, and all fluorescent or blue-stained nodes were identified as SLNs. The number and location of SLNs were documented, and each SLN was submitted separately for pathological examination.After SLN retrieval, laparoscopic hysterectomy was performed. Pelvic lymphadenectomy plus para-aortic lymph node dissection was performed in cases of failed SLN mapping, enlarged pelvic or para-aortic lymph nodes, or uterine findings on gross inspection including uterine cavity lesion involving > 1/2 of the cavity or myometrial invasion > 1/2 of the myometrial thickness. All surgical procedures were performed by the same surgical team.

#### Sentinel lymph node evaluation

According to the definitions and descriptions of SLNs detection rate and accuracy rate in the 8th edition of the AJCC Cancer Staging Manual by the American Joint Committee on Cancer (AJCC)^10^: successful tracing was defined as visualization of fluorescent lymph nodes intraoperatively with the naked eye, while identification failure referred to cases where tissue fluorescence was observed but no fluorescent lymph nodes were detected; SLN detection rate was calculated as (number of cases with detected SLNs/total number of cases in the study group) × 100%; accuracy rate was calculated as (number of pathologically positive SLNs/number of cases with intraoperatively detected SLNs) × 100%, based on the final pathological results (diagnosis by paraffin section).

Given that most enrolled patients had early-stage lesions and did not undergo systematic lymphadenectomy, the sensitivity and false-negative rate could not be calculated temporarily and would be further improved in subsequent studies.

#### Timeliness statistics

Lymph node visualization time: the time (in seconds) elapsed from the initiation ICG injection into the proper ovarian ligament to the distinct visualization of lymph nodes along the IPP pathway.Operative time was defined as the total time from the establishment of pneumoperitoneum by laparoscopic puncture to the completion of surgery and skin incision suture.Hospital stay was defined as the total number of inpatient days from the day of surgery until the patient met discharge criteria, including stable vital signs, absence of postoperative complications, satisfactory incision healing, and tolerance of normal diet and activity.IPP procedure time was defined as the time taken to complete ICG solution injection at the bilateral proper ovarian ligaments.

#### Postoperative complication statistics

Calculate the number of patients with abnormal liver and kidney functions and lymphatic fistula after surgery; the calculation formula is (number of complicated cases/total number of cases) × 100%.

#### Statistical methods

Descriptive statistical methods were adopted to summarize the baseline clinical and pathological characteristics of patients; for continuous variables such as age, body mass index (BMI) and tumor size, the median (range) was calculated and reported to reflect the central tendency and distribution range of the data; for categorical variables including tumor location, differentiation degree, lymphovascular space invasion (LVSI) and microcystic elongated and fragmented (MELF) invasion, the frequency (percentage) was calculated and reported; For time-related data, the mean and standard deviation (SD) were calculated to describe the central tendency and dispersion of the data, and the median was reported to reflect the middle position of the data. All calculations were performed using SPSS Statistics 29 and R 4.4.2, and the results were double-checked to ensure accuracy.

## Results

### Clinical and pathological characteristics of patients

A total of 32 patients were enrolled in the study. Postoperative staging showed 22 cases of stage IA, 4 cases of stage IB, 3 case of stage II, 1 case of stage IIIA, 1 case of stage IIIC1, and 1 case of stage IIIC2.

**Table 1:** Baseline Clinical and Pathological Characteristics of Patients.

Characteristic	Details
Age (years)	57(25–70)
Body Mass Index (BMI, kg/m²)	23.75 (17.33–30.73)
Tumor Size (cm)	2.3(0–9.5.5)
Tumor Location	n (%)
Left uterine cornu	6(18.8%)
Right uterine cornu	4(12.5%)
Uterine cavity	11(34.4%)
Uterine fundus	5(15.6%)
Cervical isthmus	1(3.1%)
No definite mass on gross examination	5(15.6%)
Differentiation Grade	n (%)
FIGO grade 1	7(21.9%)
FIGO grade 2	12(37.5%)
FIGO grade 3	6(18.8%)
Atypical hyperplasia	5(15.6%)
Carcinosarcoma	1(3.1%)
No lesion	1(3.1%)
Lymphovascular Space Invasion (LVSI)	n (%)
Positive	9 (28.1%)
Negative	23(71.9%)
Microcystic, Elongated, and Fragmented invasion (MELF)	n (%)
Positive	4(12.5%)
Negative	28(87.5%)

*LVSI* lymphovascular space invasion, *MELF* microcystic, elongated and fragmented invasion; continuous variables are presented as median (range), and categorical variables as number of cases (percentage).

*Lymphatic drainage pathways and common locations of SLNs:* Following tracer injection of methylene blue via the cervical route, among the 25 patients with visualized lymph nodes, 23 cases (92%) showed the UPP lymphatic drainage pathway, with SLNs located in the obturator and external iliac regions; 2 cases (8%) demonstrated the LPP pathway, with SLNs located in the common iliac region. The IPP pathway was not observed in any case. Via injection of ICG tracer into the proper ovarian ligament, only the IPP pathway was visualized; left para-aortic SLNs were commonly located between the level of the inferior mesenteric artery and renal vein, while right para-aortic SLNs were situated at a relatively lower level than the left side, easy to expose, and mostly found on the surface of the inferior vena cava approximately 2–3 cm above the aortic bifurcation. Lymphatic drainage between the left and right para-aortic regions was found to be interconnected, and no overlap was observed between the lymphatic drainage pathways of ICG and methylene blue.

*Lymph node visualization time and duration of the IPP pathway:* The shortest time to initial visualization of sentinel lymph nodes (SLNs) was 10 s, the longest was 137 s, with a median of 45 s and a mean of 50.9 s. For the left infundibulopelvic pathway (IPP), the mean time from injection to distinct lymph node visualization was 45.75 ± 22.78 s, with a median of 42.5 (18–80) seconds; for the right side, the mean time was 54.5 ± 45.89 s, with a median of 35 (10–137) seconds. Visualization duration: The visualization of para-aortic sentinel lymph nodes persisted until intraoperative resection or the end of the surgery.

*SLNs detection rate and accuracy rate:* Of the 32 patients, the detection rate of ICG-enhanced left para-aortic sentinel lymph nodes (SLNs) was 80.6% (25/31; one patient was excluded from IPP pathway injection due to severe adhesion in the left adnexal region found intraoperatively), and the detection rate of right para-aortic SLNs was 93.8% (30/32).The mean number of left para-aortic SLNs was 3.81 (range 1–8), with an accuracy of 95.8% (one case was confirmed as adipose tissue on postoperative pathology). The mean number of right para-aortic SLNs was 4.96 (range 1–12), with an accuracy of 100%.Bilateral pelvic SLNs were mainly visualized in the obturator, external iliac, and internal iliac regions. Intraoperative observation showed that the enhancement pathways of ICG and methylene blue did not overlap.

*Postoperative complication incidence rate:* Of the 32 patients, 1 patient (3.13%) developed postoperative lymphatic leakage.Preoperatively normal liver and kidney function deteriorated postoperatively in 1 patient (3.13%).These two cases involved different individuals.

*Operative time and hospital stay:* The mean operative time was 3.08 ± 0.63 h (range 2.0–4.3 h) in the dual-pathway group, and 3.56 ± 1.23 h (range 1.8–7.0 h) in the conventional surgery group, which underwent para-aortic sentinel lymph node biopsy or systematic lymphadenectomy. The duration of the IPP tracing procedure alone was 7.13 ± 4.05 min (range 2.0–16.0 min).

The mean postoperative hospital stay was 7.41 ± 2.49 days (range 4–17 days) in the dual-pathway group and 9.16 ± 3.25 days (range 4–20 days) in the conventional surgery group. There was a statistically significant difference in postoperative hospital stay between the two groups (*P* = 0.020), with the dual-pathway group demonstrating a significantly shorter hospital stay.

Notably, the dual-pathway strategy did not increase the incidence of postoperative complications, supporting its safety and clinical feasibility.

*Report of a special case:* Among the 32 patients, we identified one case of isolated para-aortic lymph node metastasis. Specifically, the pelvic lymph nodes were pathologically negative for metastasis, whereas 1 of the 7 right para-aortic sentinel lymph nodes was histologically positive. This finding indirectly demonstrates the importance of the IPP pathway as a supplementary strategy for pelvic and abdominal lymphatic mapping and tracing.

**Fig. 2:**
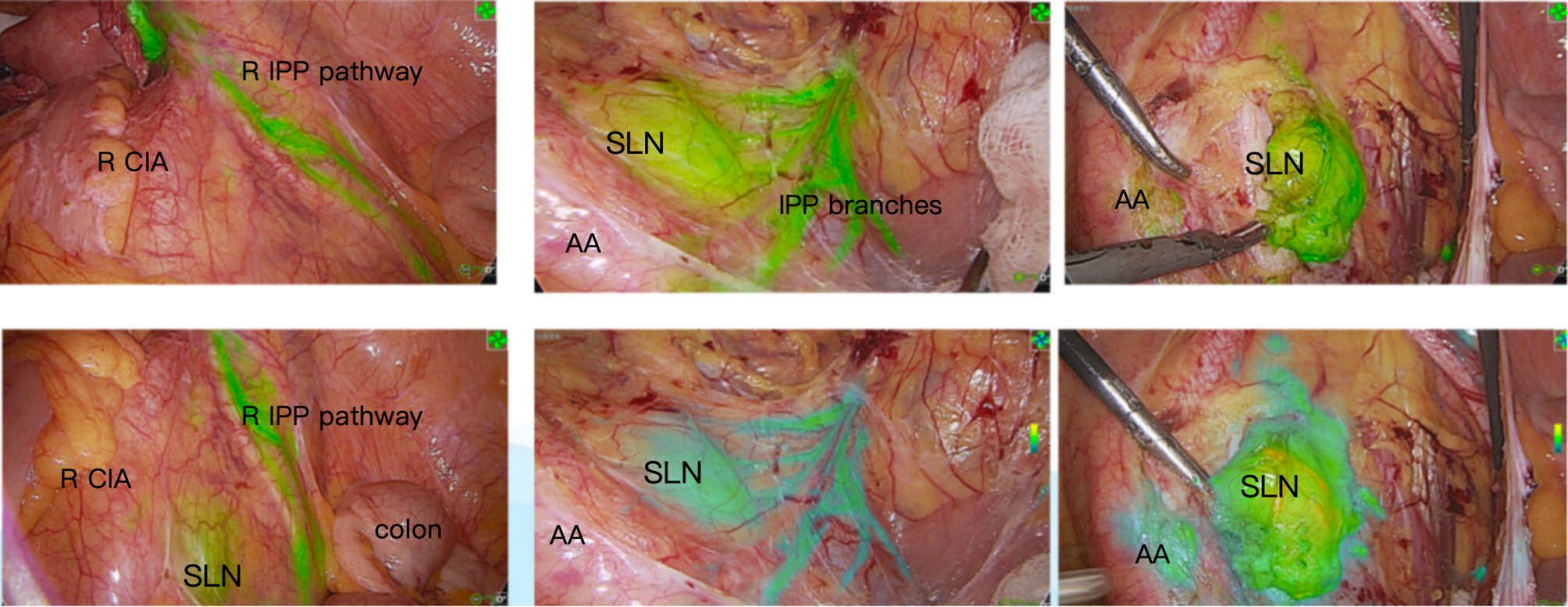
Visualization of right para-aortic sentinel lymph nodes with ICG.

**Fig. 3:**
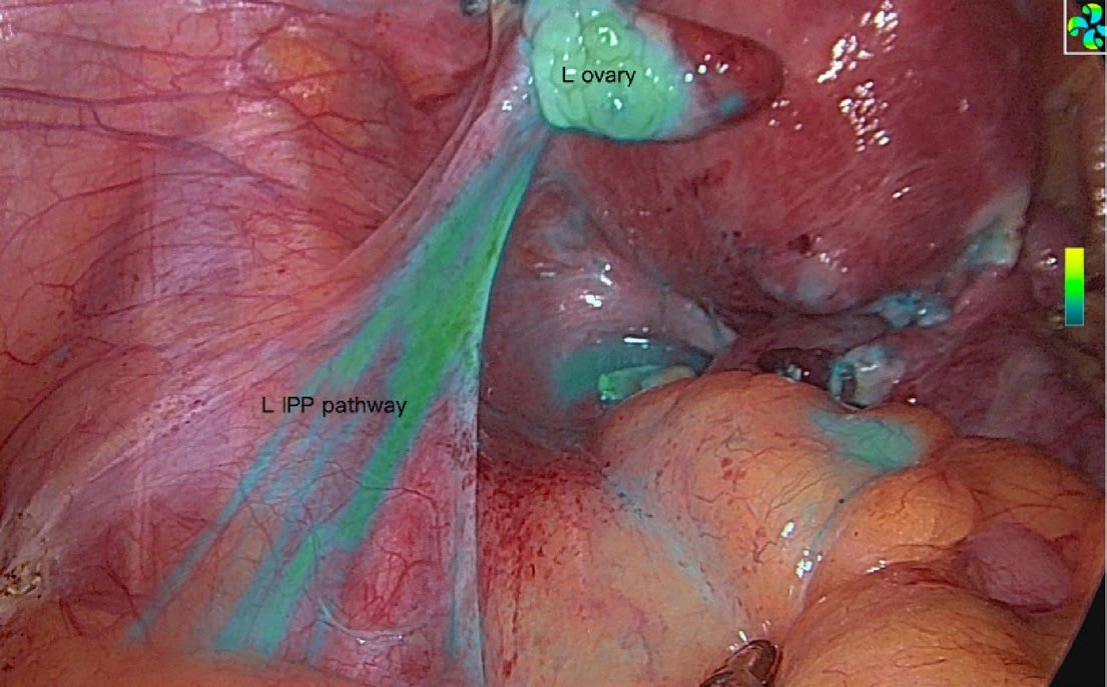
Visualization of Lymphatic Vessels in the Left IPP Pathway.

**Fig. 4:**
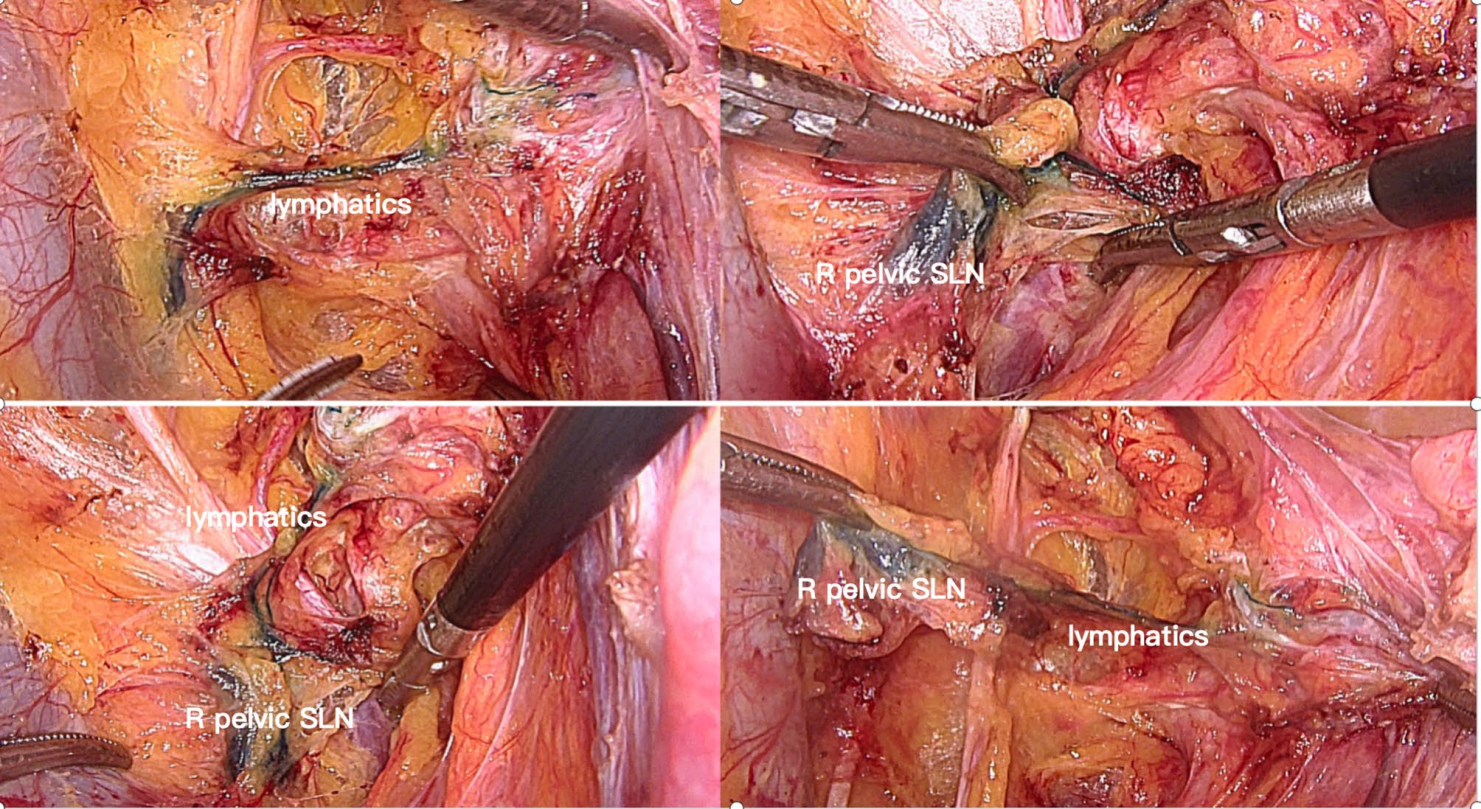
Visualization of Pelvic Sentinel Lymph Nodes with Methylene Blue.

**Fig. 5:**
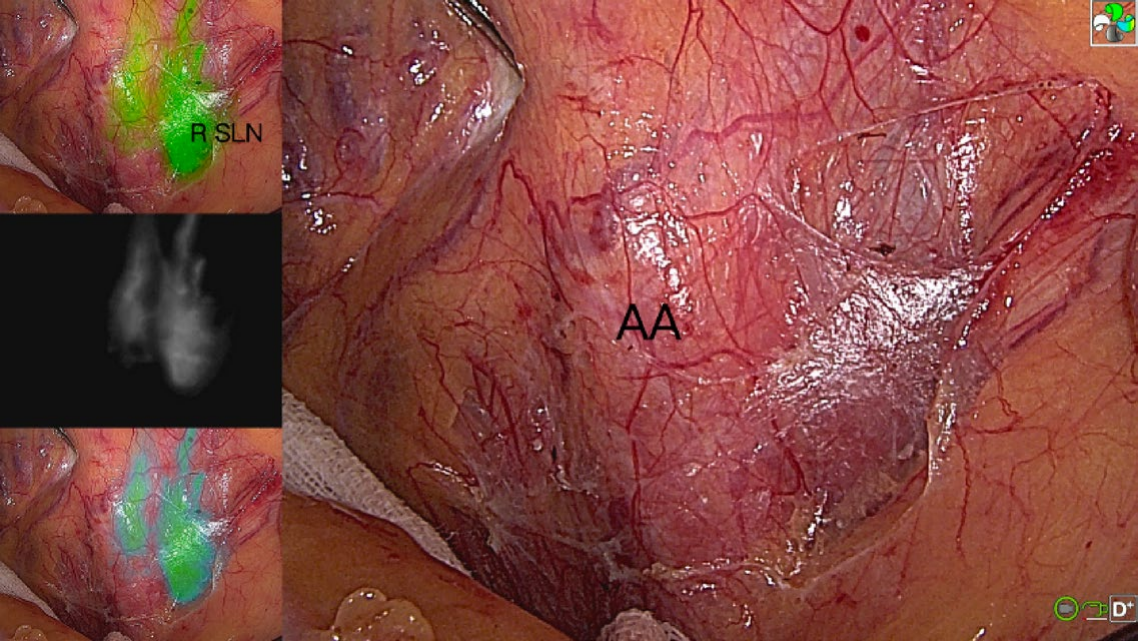
Multi-color Contrast Visualization of Para-aortic Sentinel Lymph Nodes.

**Fig. 6:**
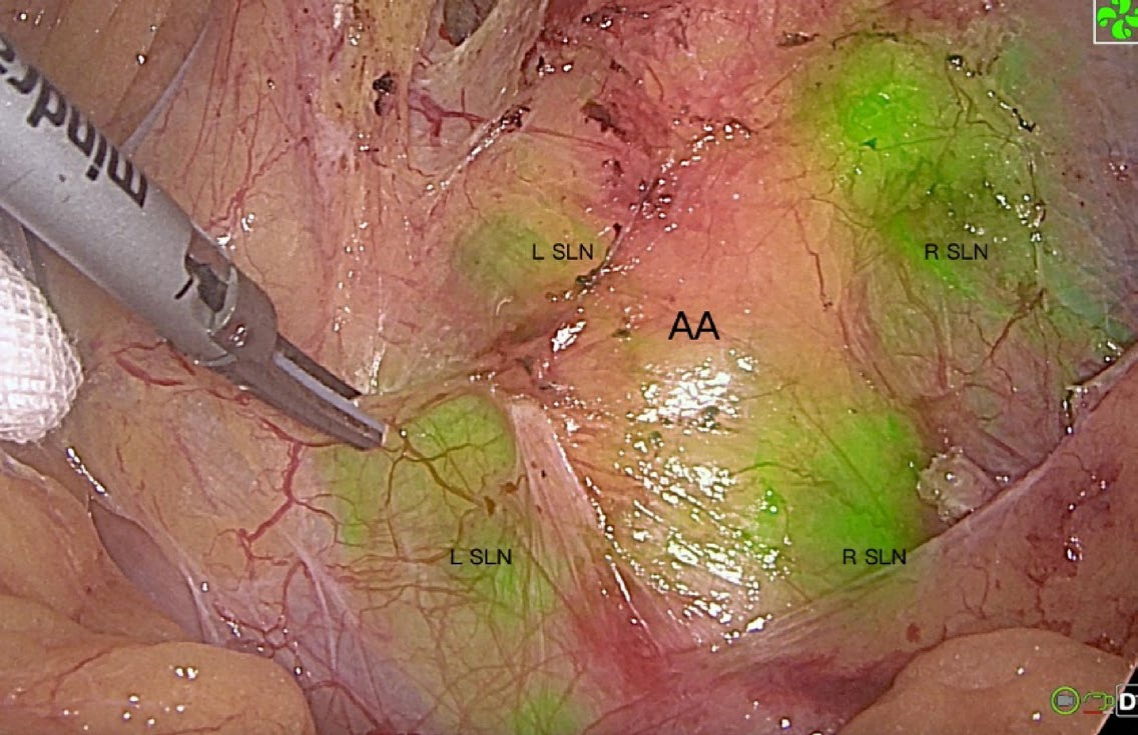
Bilateral Para-aortic SLN via ICG.

Injection of ICG into the proper ovarian ligament and lymphatic vessel visualization (see Video 1); resection of para-aortic sentinel lymph nodes (see Video 2); resection of pelvic sentinel lymph nodes (see Video 3); visualization of the right IPP pathway and its SLNs in the para-aortic region via ICG (see Fig. 2); visualization of lymphatic vessels in the left IPP pathway (see Fig. 3); visualization of pelvic SLNs via methylene blue (see Fig. 4); multi-color contrast visualization of para-aortic sentinel lymph nodes (see Fig. 5);bilateral para-aortic SLN via ICG(see Fig. 6).

## Discussion

Lymph node metastasis is a common metastatic pattern in endometrial cancer. Currently, three main lymphatic drainage pathways from the uterine corpus are recognized: The upper paracervical pathway (UPP), which drains to the medial side of the external iliac vessels and the superior obturator region; The lower paracervical pathway (LPP), which drains to the common iliac and presacral regions; The infundibulopelvic pathway (IPP), which drains to the para-aortic region.Studies have shown that lymphatic metastatic routes in endometrial cancer are related to tumor location. Tumors located at the fundus or cornua are prone to para-aortic lymph node metastasis.In fact, the location of sentinel lymph node (SLN) visualization in endometrial cancer is associated with the injection site of the tracer. For example, hysteroscopic peritumoral injection of ^99^ᵐTc yields a para-aortic SLN detection rate of 24%. Subserosal injection of blue dye and ^99^ᵐTc into the uterine corpus achieves a detection rate of up to 30%. In contrast, the detection rate of para-aortic SLNs is extremely low with cervical injection of tracers^11^.

Regardless of the method used, the detection rate of para-aortic SLNs remains relatively low. Moreover, cervical injection is the mainstream technique for SLN mapping in endometrial cancer, which has significant limitations. Especially in high-risk patients with tumors located at the fundus or cornua, the conventional cervical injection technique is inadequate to detect para-aortic SLNs with skip metastasis, potentially leading to missed high-level metastatic lesions.

In addition to the conventional cervical injection method, other approaches have been used to visualize para-aortic lymph nodes. Direct intraperitoneal injection or paravascular adipose tissue injection^12^ improves the local detection rate by injecting tracer directly into the para-aortic region. However, this method carries a high risk of vascular and nerve injury and cannot truly reflect the physiological lymphatic drainage pathway, thus limiting its clinical application.The fundal subserosal injection method^13^ is limited by low tracer coverage. The three‑point subserosal injection method^14^ shows a wide and unstable range of detection rates (61.5%–78%) and is cumbersome, prolonging operative time.Hysteroscopic endometrial injection^15^ and hysteroscopic peritumoral injection^16^ require uterine distension, which may damage the endometrium or cause tumor cell dissemination. Furthermore, the tracer is confined to the superficial endometrial layer, resulting in insufficient mapping of lymphatic drainage from the deep myometrium.Uterine myometrial injection^17^ lacks clear data on detection rate and direct evidence supporting its clinical efficacy. Precise control of injection depth is difficult: an overly superficial injection is too close to the serosa, whereas an overly deep injection approaches the endometrium, both of which compromise accurate lymphatic mapping.

The currently more recognized method is the direct infundibulopelvic ligament injection method, in which a tracer is directly injected into the infundibulopelvic ligament to specifically enhance local visualization. However, the ligament is densely populated with blood vessels and nerves, leading to high operational risks and a high chance of damaging blood vessels and nerves; it may also disrupt the lymphatic drainage structure, thus affecting the authenticity of visualization results. In this study, an innovative dual-tracer protocol of “methylene blue injection via the cervix + ICG injection via the proper ovarian ligament” was adopted. The proper ovarian ligament was selected as the ICG injection site instead of direct injection into the infundibulopelvic ligament, which can avoid damaging the abundant blood vessels and lymphatic vessels within the ligament, thereby reducing experimental errors.

Para-aortic SLNs identified by the conventional cervical injection method are mostly concentrated in the region from the common iliac bifurcation to the inferior aspect of the inferior mesenteric artery^18,19^. In this study, tracing via the IPP pathway revealed that left para-aortic SLNs were mainly located from the superior aspect of the inferior mesenteric artery to the inferior aspect of the left renal vein, while right para-aortic SLNs were concentrated on the surface of the inferior vena cava from the bilateral common iliac bifurcation to the level of the inferior mesenteric artery, covering the high-level region that is difficult to reach with traditional methods.

**Fig. 7:**
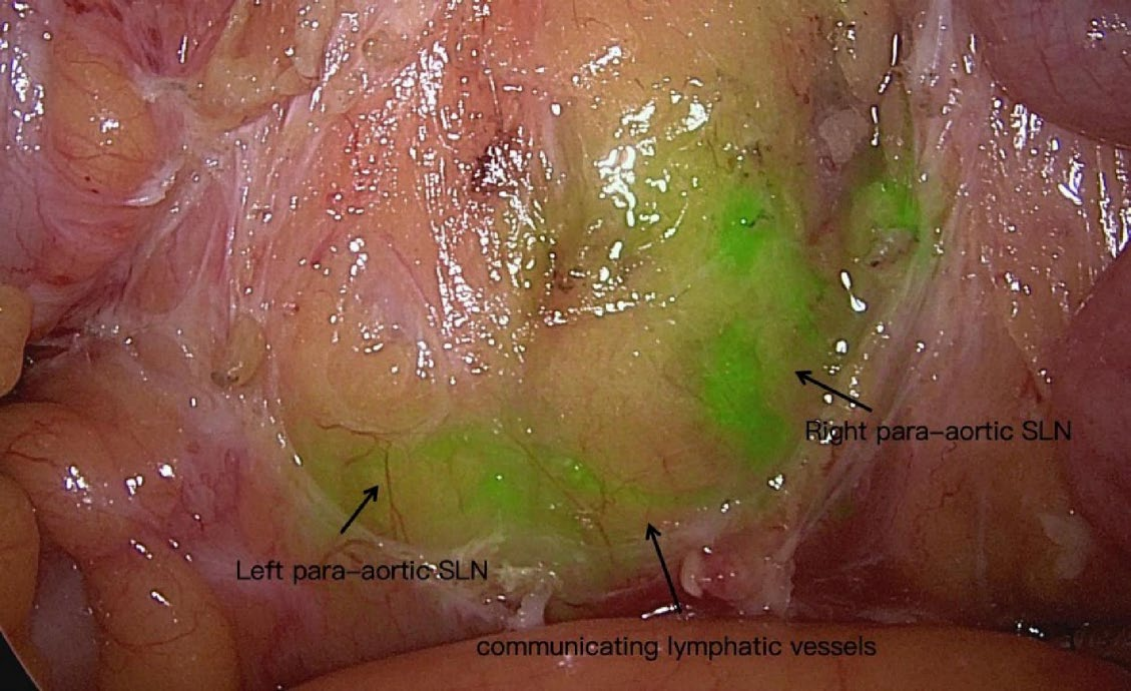
Visualization of Left Para-aortic Sentinel Lymph Nodes via Lymphatic Anastomoses with ICG.

In terms of success rate, a systematic review by Cormier et al. showed that the detection rates of para-aortic lymph nodes after conventional cervical injection and deep cervical injection of contrast agents were only 2% and 17%, respectively^20^. In this study, the detection rate of para-aortic SLNs reached up to 93.8% with an accuracy of 100%, which was significantly superior to the traditional cervical injection method.Furthermore, it is even feasible to inject the tracer only through the unilateral IPP pathway, and the contralateral SLNs can be visualized through the communicating branches of the lymphatic pathway, which greatly improves the success rate of visualization(see Fig. 7).In terms of speed, the average visualization time of left para-aortic SLNs in this study was 45.75 s, and that of right para-aortic SLNs was 54.5 s, which was much faster than the tens of minutes required by traditional methods^21^.

In addition, the “dual tracer + dual pathway” design of this study has significant innovation. There was no overlapping visualization between methylene blue injected via the cervical route and indocyanine green (ICG) injected via the IPP route. This design not only enables clear identification of pelvic SLNs through methylene blue but also captures para-aortic SLNs via the IPP pathway through ICG, allowing comprehensive visualization of pelvic and para-aortic sentinel lymph nodes in endometrial cancer.

**Fig. 8:**
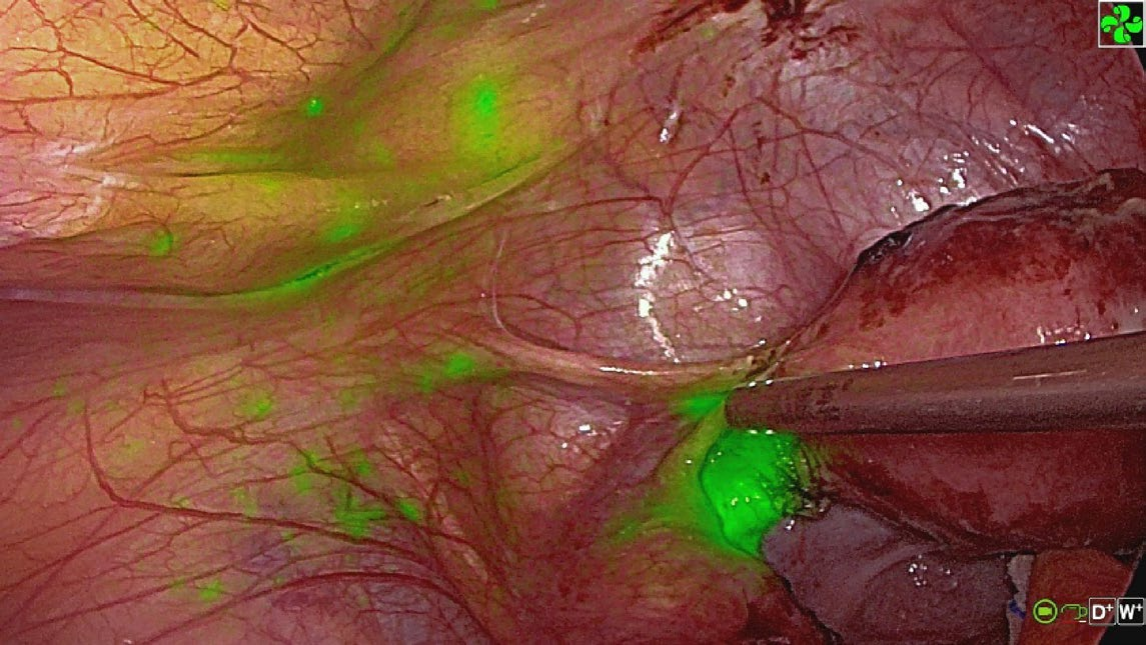
“Starry-sky” Phenomenon.

During the tracer operation of the IPP pathway, standardized procedures must be strictly followed to ensure efficacy. For the injection step, the concentration of ICG should be strictly controlled at 0.125 mg/ml (this concentration has been verified by repeated experiments to yield the optimal visualization effect); the injection depth should be maintained at 1–2 mm with slow injection over at least 1 min; before injection, aspiration should be performed to confirm the absence of blood return, thus preventing intravascular misinjection which would cause background staining; after injection, local compression should be applied for 5–10 s, and laparoscopic gauze should be used to absorb extravasated solution to avoid non-specific staining interfering with the surgical field. For the intraoperative observation step, timing should start immediately after ICG injection, with focused tracking of the visualization direction of lymphatic vessels; the first visualized lymph node should be designated as the SLN; if obscured by intestinal loops, gentle dissection should be performed to avoid damaging the lymphatic drainage structure of the IPP pathway; for cases with unclear visualization, an additional 0.1–0.2 ml of ICG may be administered as appropriate, while excessive injection that induces background staining must be avoided. The main factors affecting SLN visualization are as follows: first, injection-related issues, including intravascular misinjection of ICG, insufficient injection volume, or excessive injection depth that penetrates the ligament and causes ICG extravasation, all of which can impair visualization efficacy; second, anatomical abnormalities, as adhesions of the ovary and fallopian tube may hinder ICG diffusion, and the injection site can be adjusted intraoperatively according to the adhesion status; third, the patient’s baseline conditions, as patients with a recent history of pelvic surgery or inflammation may present with lymphatic hyperplasia, obstruction or collateral circulation, leading to a “starry-sky” pattern of visualization (see Fig. 8), such patients require prolonged observation time and it is difficult to identify the true SLNs; fourth, equipment and reagent-related factors, as a high-resolution laparoscopic system should be used to clearly identify the fluorescent visualization of ICG, and ICG must be prepared and used immediately.

This study still has certain limitations. Firstly, the sample size is small and the research is single-centered, so the results may have selection bias and lack verification by multi-centered and large-sample data. Secondly, most of the enrolled patients had early-stage lesions and did not undergo systematic lymphadenectomy, so it is impossible to calculate the sensitivity and false-negative rate of SLN tracing for the time being, making it difficult to comprehensively evaluate its diagnostic efficacy. In addition, the research on tracer optimization is insufficient, and the effects of ICG have not been compared with those of other tracers such as nanocarbon and technetium colloid. Future research needs to be advanced from three aspects: first, expand the sample size to carry out multi-centered studies to verify the applicability of this technology in endometrial cancer with different stages and pathological types; second, explore the optimal type, injection site and timing of tracers, and formulate unified operating specifications and evaluation criteria; third, pathological ultrastaging is a key component for improving the accuracy of lymph node metastasis diagnosis. In subsequent studies, we will refine the pathological testing process, fully implement SLN pathological ultrastaging, further improve the research data, and enhance the clinical reference value of the results.

## Conclusion

The dual-pathway strategy, which involves injecting different tracers via the cervix and proper ovarian ligament respectively to visualize pelvic and para-aortic sentinel lymph nodes separately, is preliminarily proven feasible for endometrial cancer surgery in this study. It initially demonstrates the potential to facilitate a more comprehensive assessment of lymph node metastasis status in endometrial cancer patients. This strategy provides a preliminary viable approach for exploring precise treatment regimens for endometrial cancer, with the potential to reduce surgical complications and improve patients’ quality of life in future clinical practice. As a novel technique worthy of further investigation, its actual clinical value and application prospects await further verification by studies with larger sample sizes, so as to ensure the scientificity and prudence of the conclusion.

## Supplementary Information

Below is the link to the electronic supplementary material.

Supplementary Material 1

## Data Availability

The data that support the findings of this study are available from the corresponding author upon reasonable request. All original data have been properly archived in a password-protected database at the Affiliated Cancer Hospital of Guangxi Medical University, ensuring compliance with relevant ethical and privacy regulations. De-identified datasets may be shared to facilitate reproducibility, provided that appropriate institutional review board approval is obtained.
