# Supplementary figures and images for: Preliminary study on the intraoperative application of the “dual-path” strategy for sentinel lymph node tracing in endometrial cancer
Source: Sci Rep. 2026 May 11;16:21563. doi: 10.1038/s41598-026-48295-8 (PMC13350832; doi:10.1038/s41598-026-48295-8)

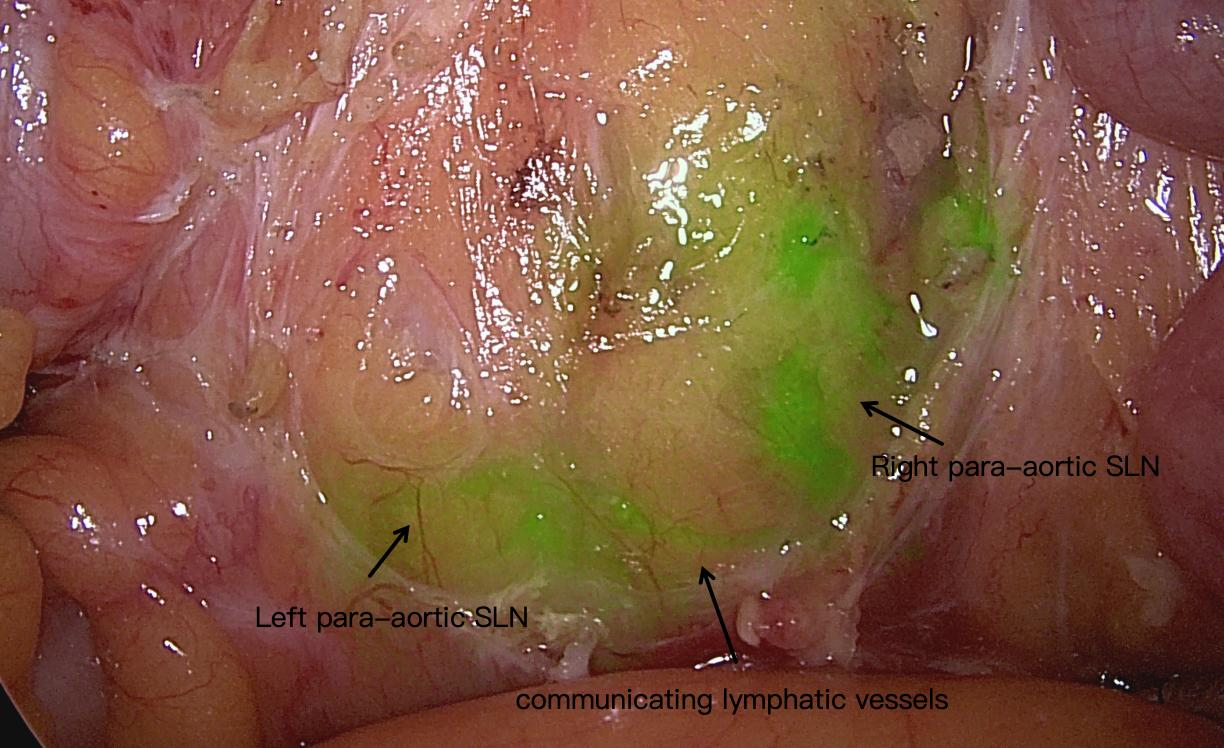

Supplement: Supplementary file 1 — Supplementary Material 1 [file 41598_2026_48295_MOESM1_ESM.png]
